# Persistance de la membrane pupillaire bilatérale et forte myopie

**DOI:** 10.11604/pamj.2018.29.222.14297

**Published:** 2018-04-23

**Authors:** Nouha Zerkaoui, Amina Laghmari

**Affiliations:** 1 Université Mohammed V Souissi, Service d’Ophtalmologie A de l’Hôpital des Spécialités de Rabat, Centre Hospitalier Universitaire Rabat, Maroc

**Keywords:** Persistance de la membrane pupillaire, forte myopie, amblyopie, agent mydriatique, Persistent pupillary membrane, high myopia, mydriatic agents

## Abstract

After birth, remnants of pupillary membrane which constitute the vascular supply to the crystalline lens can persist. These are contiguous to the iris collarette causing possible deprivation amblyopia by obstructing the pupillary area. We here report the case of a 4-year-old child, born to consanguineous parents, presenting with bilateral persistent pupillary membrane associated with high myopia. Clinical examination showed bilateral remnants of pupillary membrane, intact iris sphincter and diffuse chorioretinal atrophy in the fundus of eye . Visual acuity was difficult to assess. On the other hand, refraction test showed high myopia RE -10.75 (-3.25, 29°) LE -10 (-0.75, 180°). Treatment of bilateral persistent pupillary membrane is based on mydriatic agents, surgical excision or laser destruction. We opted for the use of mydriatic agents with total optical correction and amblyopia treatment.

## Image en médecine

Après la naissance, des vestiges de la membrane pupillaire qui constituait l'apport vasculaire pour le cristallin peuvent persister. Ces vestiges restent accolés à la collerette irienne pouvant causer une amblyopie par privation en obstruant l'aire pupillaire. Nous allons rapporter le cas d'un enfant de 4 ans, de parents consanguins et qui présente une persistance de la membrane pupillaire bilatérale associée à une forte myopie. Son examen a montré des vestiges de la membrane pupillaire bilatérale, un sphincter irien intact et au fond d'œil une atrophie chorio rétinienne diffuse. L'acuité visuelle était difficile à apprécier, la réfraction par contre a montré l'existence d'une forte myopie OD -10.75 (-3.25 A 29°) OG -10 (-0.75 A 180°). La gestion de la persistance de la membrane pupillaire peut se faire par l'utilisation d'agents mydriatiques, par excision chirurgicale ou par destruction au laser. Pour notre cas nous avons opté pour l'utilisation d'agents mydriatiques avec prescription de correction optique totale et traitement de l'amblyopie.

**Figure 1:**
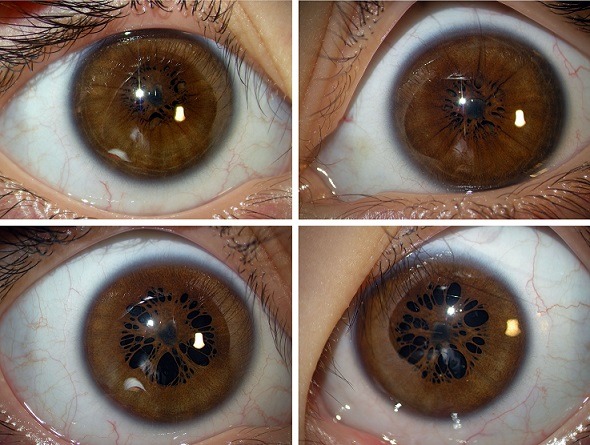
persistance de la membrane pupillaire bilatérale en ODG avant et après dilatation

